# CPVT-associated cardiac ryanodine receptor mutation G357S with reduced penetrance impairs Ca^2+^ release termination and diminishes protein expression

**DOI:** 10.1371/journal.pone.0184177

**Published:** 2017-09-29

**Authors:** Yingjie Liu, Jinhong Wei, Siobhan M. Wong King Yuen, Bo Sun, Yijun Tang, Ruiwu Wang, Filip Van Petegem, S. R. Wayne Chen

**Affiliations:** 1 Libin Cardiovascular Institute of Alberta, Department of Physiology and Pharmacology, University of Calgary, Calgary, Alberta, Canada; 2 Department of Biochemistry and Molecular Biology, University of British Columbia, 2350 Health Sciences Mall, Vancouver, Canada; University of Canberra, AUSTRALIA

## Abstract

Catecholaminergic polymorphic ventricular tachycardia (CPVT) is one of the most lethal inherited cardiac arrhythmias mostly linked to cardiac ryanodine receptor (RyR2) mutations with high disease penetrance. Interestingly, a novel RyR2 mutation G357S discovered in a large family of more than 1400 individuals has reduced penetrance. The molecular basis for the incomplete disease penetrance in this family is unknown. To gain insights into the variable disease expression in this family, we determined the impact of the G357S mutation on RyR2 function and expression. We assessed spontaneous Ca^2+^ release in HEK293 cells expressing RyR2 wildtype and the G357S mutant during store Ca^2+^ overload, also known as store overload induced Ca^2+^ release (SOICR). We found that the G357S mutation reduced the percentage of RyR2-expressing cells that showed SOICR. However, in cells that displayed SOICR, G357S reduced the thresholds for the activation and termination of SOICR. Furthermore, G357S decreased the thermal stability of the N-terminal domain of RyR2, and markedly reduced the protein expression of the full-length RyR2. On the other hand, the G357S mutation did not alter the Ca^2+^ activation of [^3^H]ryanodine binding or the Ca^2+^ induced release of Ca^2+^ from the intracellular stores in HEK293 cells. These data indicate that the CPVT-associated G357S mutation enhances the arrhythmogenic SOICR and reduces RyR2 protein expression, which may be attributable to the incomplete penetrance of CPVT in this family.

## Introduction

Catecholaminergic polymorphic ventricular tachycardia (CPVT) is an inherited, life-threatening cardiac arrhythmia characterized by stress- and emotion-induced bidirectional or polymorphic ventricular tachycardia and sudden death. Interestingly, patients susceptible to CPVT generally have structurally normal hearts and normal electrocardiograph (ECG) at rest [1]. Genes encoding the cardiac ryanodine receptor (RyR2), cardiac calsequestrin (CASQ2), cardiac triadin, and calmodulin have been linked to CPVT [1–7]. Most CPVT cases are associated with mutations in RyR2 [1]. A major focus of investigation over the past decade is to understand how mutations in RyR2 cause CPVT. An increasing body of evidence indicates that CPVT is caused by delayed afterdepolarizations (DADs) and triggered activities [1, 8, 9]. DADs are the result of increased activity of the Na^+^/Ca^2+^ exchanger in response to spontaneous diastolic Ca^2+^ release from the sarcoplasmic reticulum (SR) [10–12]. Hence, spontaneous SR Ca^2+^ release is a well-known cause of CPVT.

It has long been recognized that spontaneous SR Ca^2+^ release occurs in the form of Ca^2+^ waves in cardiac cells when either the SR Ca^2+^ load exceeds a threshold level (the SR Ca^2+^ activation threshold) or the threshold is reduced to a level below the SR Ca^2+^ load [13–15]. In light of its dependence on the SR Ca^2+^ load and the SR Ca^2+^ activation threshold, we referred to this spontaneous SR Ca^2+^ release as store-overload induced Ca^2+^ release (SOICR) [1, 16, 17]. In support of the link between spontaneous SR Ca^2+^ release and CPVT, RyR2 mutations associated with CPVT were found to enhance the propensity for spontaneous Ca^2+^ release by lowering the activation threshold for SOICR [1, 8, 16–20]. Some CPVT RyR2 mutations also increased the amplitude of spontaneous Ca^2+^ release by delaying the termination of SOICR or lowering the termination threshold for SOICR [21]. Thus, these studies support the notion that the activation and termination thresholds for SOICR are important determinants of CPVT susceptibility.

CPVT is one of the most lethal cardiac arrhythmias occurring mainly in young people with sudden death often being the first symptom of CPVT patients. The mean age of onset of symptom in subjects with RyR2 mutations is ~17 years old. Furthermore, many CPVT-linked RyR2 mutations (~20%) are de novo in origin [22]. Because of the highly lethal nature of CPVT-linked RyR2 mutations, most families with CPVT RyR2 mutations are relatively small and show high penetrance (50–90%) [23–26]. Interestingly, a novel RyR2 mutation G357S has recently been discovered in a large family with 10 generations and 1404 members [27]. There are 179 G357S mutant carriers, 36 of which suffered from sudden cardiac death. Despite this lethal outcome to ~20% of the G357S positive subjects, many G357S mutant carriers lived beyond the fifth decade and even to their 70–80’s [27]. This raises an important and interesting question of why the disease expression of the RyR2 mutation G357S is highly variable in this family.

The G357S mutation is located in the N-terminal region of the RyR2 channel, which consists of three individual domains: domain A (residues 1–217), domain B (residues 218–409), and domain C (residues 410–631). This region forms a tetrameric gating ring that undergoes large motions during channel opening [28–31]. Interestingly, deletion of individual domains has uncovered distinct roles in RyR2 function [32]. For instance, domain B, where the G357S mutation is located, is believed to be important for RyR2 protein expression and the activation and termination of SOICR [32]. Surprisingly, it was showed that the G357S mutation exerted little effect on the propensity for SOICR in HEK293 cells [27]. However, upon treatment with forskolin which activates PKA, the G357S-expressing HEK293 cells did display enhanced SOICR activity compared to WT expressing cells treated with forskolin [27]. These observations indicate that, unlike other N-terminal CPVT RyR2 mutations that display enhanced SOICR activity in the absence of PKA activation, the effect of the G357S mutation on SOICR requires PKA-dependent phosphorylation. It is proposed that this dependence on sympathetic activation of the effect of the G357S mutation may contribute to the reduced penentrance (28%) of CPVT in the G357S mutant carrieres who may experience varied levels of sympathetic activation [27]. However, in addition to altering the response to PKA activation, it remains to be determined whether the G357S mutation could affect other properties of the RyR2 channel.

Considering the roles of domain B in RyR2 function, in the present study, we assessed the impact of the G357S mutation on the properties of SOICR and the protein expression of RyR2. We found that although the G357S mutation had little effect on the propensity for SOICR, which is consistent with that reported previously [27], it significantly reduced the activation and termination threshold for SOICR. Furthermore, the G357S mutation decreased the thermal stability of the the N-terminal domain of RyR2, and dramatically reduced the expression of the full-length RyR2 protein. However, the G357S mutation did not alter the cytosolic Ca^2+^-dependent activation of [^3^H]ryanodine binding or the cytosolic Ca^2+^ regulated Ca^2+^ release in HEK293 cells. The markedly reduced protein expression of the G357S mutant may contribute to the reduced disease penetrance in this large family.

## Materials and methods

### Construction of the RyR2-G357S mutation

The point mutation G357S in mouse RyR2 was generated by the overlap extension method using PCR [33, 34]. Briefly, a NheI/AflII fragment with mutation G357S was obtained by overlapping PCR. The NheI/AflII fragment was removed from the PCR product and was used to replace the corresponding WT fragment in the full-length RyR2 cDNA in the expression plasmid pcDNA5. The mutation and sequence of the PCR product were confirmed by DNA sequencing.

### Generation of stable inducible HEK293 cell lines and cell culture

Stable inducible HEK293 cell lines expressing RyR2 WT or the G357S mutant were generated using the Flp-In T-REx Core Kit from Invitrogen. Briefly, Flp-In T-REx-293 cells were co-transfected with the inducible expression vector pcDNA5/FRT/TO containing the RyR2 WT or mutant cDNA and the pOG44 vector encoding the Flp recombinase in 1:4 ratios using the Ca^2+^ phosphate precipitation method. The transfected cells were washed with PBS (phosphate buffered saline: 137 mM NaCl, 8 mM Na_2_HPO_4_, 1.5 mM KH_2_PO_4_ and 2.7 mM KCl, pH 7.4) 24 h after transfection followed by a change to fresh medium for 24 h. The cells were then washed again with PBS, harvested and plated onto new dishes. After the cells had attached (~4 h), the growth medium was replaced with a selection medium containing 200 μg/ml hygromycin (Invitrogen). The selection medium was changed every 3–4 days until the desired number of cells was grown. The hygromycin-resistant cells were pooled, aliquoted (1 ml) and stored at −80°C. These positive cells are believed to be isogenic because the integration of RyR2 cDNA is mediated by the Flp recombinase at a single FRT site. HEK293 cells were maintained in Dulbecco’s modified Eagle’s medium (DMEM) supplemented with 0.1 mM minimum Eagle’s medium nonessential amino acids, 4 mM L-glutamine, 100 units of penicillin / ml. 100 mg of streptomycin/ml, 4.5 g of glucose/ liter, and 10% fetal calf serum, at 37 ^o^C under 5% CO_2_.

### Immunoblotting

HEK293 cell lines grown for 24 h after induction by 1 μg/ml tetracycline (Sigma) were washed with PBS plus 2.5 mM EDTA and harvested in the same solution by centrifugation for 8 min at 700 g in an IEC Centra-CL2 centrifuge. The cells were then washed with PBS without EDTA and centrifuged again at 700 g for 8 min. The PBS-washed cells were solubilized in a lysis buffer containing 25 mM Tris, 50 mM Hepes (pH 7.4), 137 mM NaCl, 1% CHAPS, 0.5% soya bean phosphatidylcholine, 2.5 mM DTT and a protease inhibitor mix (1 mM benzamidine, 2 μg/ml leupeptin, 2 μg/ml pepstatin A, 2 μg/ml aprotinin and 0.5 mM PMSF). This mixture was incubated on ice for 1 h. Cell lysates were obtained by centrifuging twice at 16000 g in a microcentrifuge at 4°C for 30 min to remove unsolubilized materials. The RyR2 WT and G357S mutant proteins were subjected to SDS/PAGE (6% gel) and transferred onto nitrocellulose membranes at 100 V for 2 h at 4°C in the presence of 0.01% SDS [35, 36]. The nitrocellulose membranes containing the transferred proteins were blocked for 45 min with 1x PBS and 1% Casein Blocker (Bio-Rad). The blocked membrane was incubated with the anti-RyR antibody (34C) (Thermo Scientific, MA3-925, 1:1000 dilution) and then incubated with the secondary anti-[mouse IgG (heavy and light)] antibodies conjugated to horseradish peroxidase (1:20,000 dilution). For β-actin determination, the blocked membrane was incubated with the anti-β-actin antibody (1:5000) and then incubated with the same secondary antibody. After washing for 5 min three times, the bound antibodies were detected using an enhanced chemiluminescence kit from Pierce.

### Single-cell cytosolic Ca^2+^ imaging of HEK293 cells

Cytosolic Ca^2+^ levels in stable inducible HEK293 cells expressing RyR2 WT or G357S were monitored using single cell Ca^2+^ imaging and the fluorescent Ca^2+^ indicator dye Fura-2 AM (Fura-2 acetoxymethyl ester) as described previously [16, 17]. Briefly, cells grown on glass coverslips for 18–22 h after induction by 1 μg/ml tetracycline were loaded with 5 μM Fura-2 AM in KRH (Krebs–Ringer–Hepes) buffer (125 mM NaCl, 5 mM KCl, 1.2 mM KH_2_PO_4_, 6 mM glucose, 1.2 mM MgCl_2_ and 25 mM Hepes, pH 7.4) plus 0.02% pluronic F-127 and 0.1 mg/ml BSA for 20 min at room temperature (23°C). The coverslips were then mounted in a perfusion chamber (Warner Instruments) on an inverted microscope (Nikon TE2000-S). The cells were perfused continuously with KRH buffer containing increasing extracellular Ca^2+^ concentrations (0, 0.1, 0.2, 0.3, 0.5, 1.0, and 2.0 mM). Caffeine (10 mM) was applied at the end of each experiment to confirm the expression of active RyR2 channels. Time-lapse images (0.25 frame/s) were captured and analyzed with Compix Simple PCI 6 software. Fluorescence intensities were measured from regions of interest centered on individual cells. Only cells that responded to caffeine were analyzed. The filters used for Fura 2 imaging were λex = 340 ± 26 nm and 387 ± 11 nm, and λem = 510 ± 84 nm with a dichroic mirror (410 nM).

### Single-cell luminal Ca^2+^ imaging of HEK293 cells

Luminal Ca^2+^ levels in HEK293 cells expressing RyR2-WT or G357S were measured using single-cell Ca^2+^ imaging and the FRET (fluorescence resonance energy transfer)-based ER luminal Ca^2+^-sensitive cameleon protein D1ER as described previously [19, 37]. The cells were grown to 95% confluence in a 75 cm^2^ flask, passaged with PBS and plated in 100-mm-diameter tissue culture dishes at ~40% confluence 18–20 h before transfection with D1ER cDNA using the calcium phosphate precipitation method. After transfection for 24 h, the growth medium was then changed to an induction medium containing 1 μg/ml tetracycline (Sigma). After induction for ~22 h, the cells were perfused continuously with KRH buffer containing various concentrations of Ca^2+^ (0, 1 and 2 mM) and tetracaine (1 mM) for estimating the store capacity or caffeine (20 mM) for estimating the minimum store level by depleting the ER Ca^2+^ stores at room temperature (23°C). In permeabilized cells studies, the cells were first permeabilized with 50 μg/ml saponin in an incomplete intracellular-like medium (125 mM KCl, 19 mM NaCl, and 10 mM HEPES, pH 7.4 with KOH) at room temperature (23°C) for 3–4 min [38]. The cells were then switched to a complete intracellular-like medium (incomplete intracellular-like medium plus 2 mM ATP, 2 mM MgCl_2_, 0.05 mM EGTA, and 100 nM free Ca^2+^, pH7.4 with KOH) for 5–6 min to remove saponin. The permeabilized cells were then perfused with various concentrations of Ca^2+^ (0.1, 0.2, 0.4, 1, and 10 μM) followed by tetracaine (1 mM) for estimating the store capacity and caffeine (20 mM) for estimating the minimum store level by depleting the ER Ca^2+^ stores. Images were captured with Compix Simple PCI 6 software every 2s using an inverted microscope (Nikon TE2000-S) equipped with an S-Fluor 20×/0.75 objective. The filters used for D1ER imaging were λex = 436±20 nm for CFP and λex = 500±20 nm for YFP, and λem = 465±30 nm for CFP and λem = 535±30 nm for YFP with a dichroic mirror (500 nm). The amount of FRET was determined from the ratio of the light emission at 535 and 465 nm.

### [^3^H]Ryanodine binding

HEK293 cell lines grown for 24 h after induction by 1 μg/ml tetracycline (Sigma) were washed with PBS plus 2.5 mM EDTA and harvested in the same solution by centrifugation for 8 min at 700 g in an IEC Centra-CL2 centrifuge. The cells were then washed with PBS without EDTA and centrifuged again at 700 g for 8 min. The PBS-washed cells were solubilized in a lysis buffer containing 25 mM Tris, 50 mM Hepes (pH 7.4), 137 mM NaCl, 1% CHAPS, 0.5% soya bean phosphatidylcholine, 2.5 mM DTT and a protease inhibitor mix (1 mM benzamidine, 2 μg/ml leupeptin, 2 μg/ml pepstatin A, 2 μg/ml aprotinin and 0.5 mM PMSF). This mixture was incubated on ice for 1 h. Cell lysates were obtained by centrifuging twice at 16000 g in a microcentrifuge at 4°C for 30 min to remove unsolubilized materials. Equilibrium [^3^H]ryanodine binding to cell lysates was performed as described previously [39] with some modifications. [^3^H]Ryanodine binding was carried out in a total volume of 300 μl binding solution containing 30 μl of cell lysate, 500 mM KCl, 25 mM Tris, 50 mM Hepes (pH 7.4), 5 nM [^3^H]ryanodine and CaCl_2_ to set free [Ca^2+^] from pCa 9.89 to pCa 4 and a protease inhibitor mix at 37°C for 20 min. The Ca^2+^/EGTA ratio was calculated using the computer program described by Fabiato and Fabiato [40]. The binding mix was diluted with 5 ml of ice-cold washing buffer containing 25 mM Tris/HCl, pH 8.0, and 250 mM KCl and immediately filtered through Whatman GF/B filters presoaked with 1% polyethyleneimine. The filters were washed three times, and the radioactivity associated with the filters was determined by liquid scintillation counting. Non-specific binding was determined by measuring [^3^H]ryanodine binding in the presence of 50 μM unlabelled ryanodine. Maximum [^3^H]ryanodine binding was determined by measuring binding in the presence of 100 μM free Ca^2+^, 2.5 mM ATP, and 2.5mM caffeine. All binding assays were performed in duplicate.

### Expression and purification of the N-terminal RyR2ABC mutant protein

Mouse RyR2 (residues 1–547 = RyR2ABC), harboring the G357S mutation, was purified using similar procedures as described before for WT RyR2ABC [41]. In brief, the protein was expressed in the E. coli Rosetta strain as a fusion protein containing an N-terminal His-tag, maltose binding protein, and a cleavage site for Tobacco Etch Virus (TEV) Protease. After affinity steps using Nickel and amylose resin, the tag was removed with recombinant TEV protease, and further purified using Nickel, anion exchange, and size exclusion chromatography.

### Thermal melt assays

Reaction mixtures consisting of a final protein concentration of 0.3mg/mL (in 150mM KCl, 10mM HEPES, pH 7.4) and a 5,000x dilution of SYPRO® Orange Protein Gel Stain (Life Technologies) were assembled in MicroAmp® Fast Optical 48-well reaction plates (Applied Biosystems) and sealed with optical adhesive covers (Applied Biosystems). The plate was placed into a StepOnePlus Real Time PCR System (Applied Biosystems) and heated from 25°C to 95°C with a change in temperature of 0.5°C per cycle. For each construct, three replicates were performed. The resulting data were normalized, averaged and graphed using GraphPad Prism5. The first derivatives were generated to calculate the melting temperatures. Statistical significance was determined using an unpaired two-tailed t-test with a 95% confidence level.

### Statistical analysis

All values shown are mean ± SEM unless indicated otherwise. To test for differences between groups, we used Student's *t*-test (2-tailed). A *P* value <0.05 was considered to be statistically significant.

## Results

### Effect of the CPVT-associated RyR2 G357S mutation on the propensity for store-overload induced Ca^2+^ release (SOICR)

**Fig 1:**
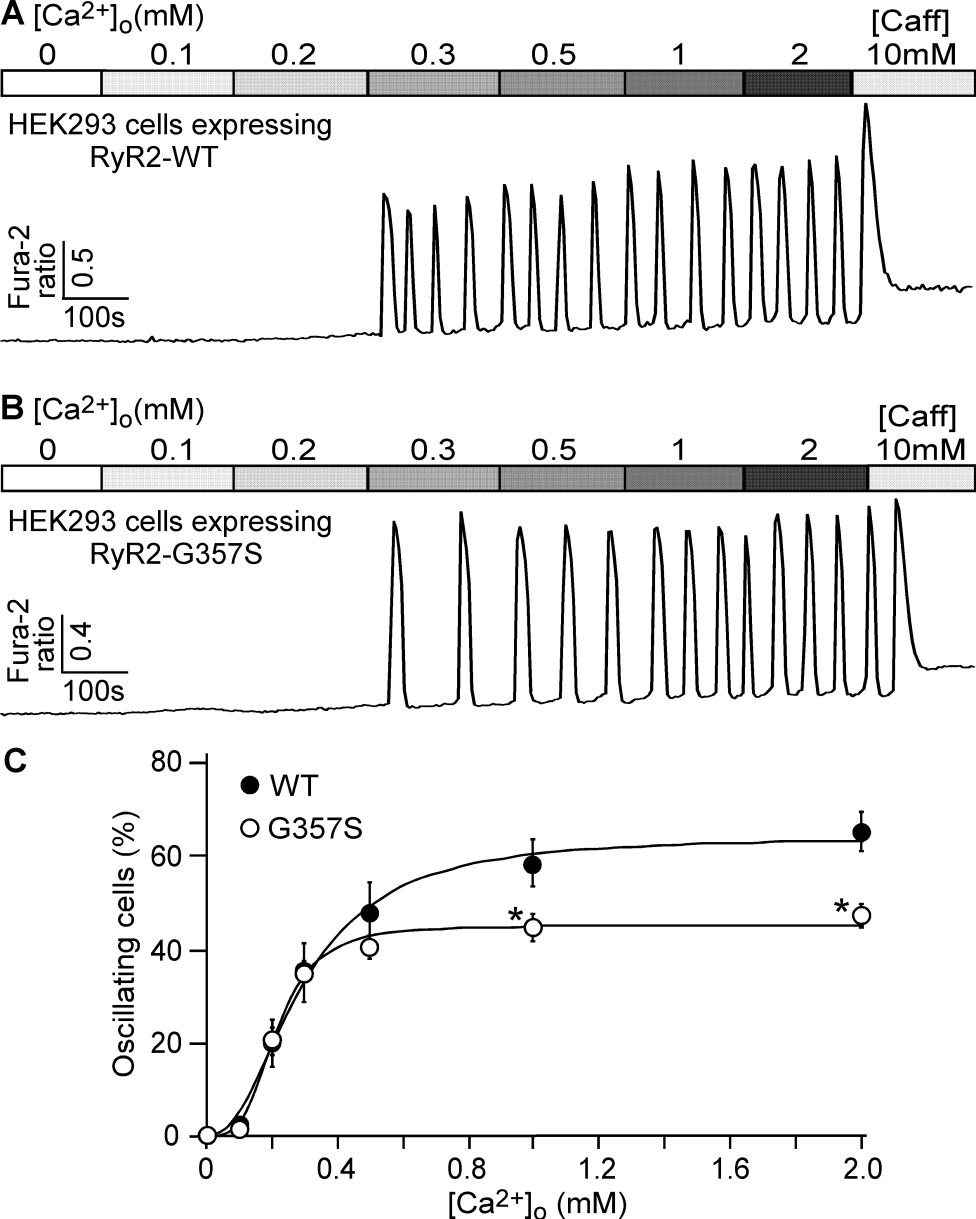
Effect of the CPVT-associated RyR2 G357S mutation on the propensity for store overload-induced Ca^2+^ release (SOICR). Stable, inducible HEK-293 cells expressing RyR2 WT (A) or RyR2 G357S (B) were loaded with 5μM Fura-2 AM in KRH buffer. The cells were then perfused continuously with KRH buffer containing increasing levels of extracellular Ca^2+^ (0–2 mM) to induce SOICR. Fura-2 ratios were recorded using epifluorescence single cell Ca^2+^ imaging. C, the percentage of RyR2 WT (898 cells) and the G357S mutant (846 cells) cells that displayed Ca^2+^ oscillations at various extracellular Ca^2+^ concentrations (n = 7). Data shown are mean ± SEM (*, p<0.01 vs WT; NS, not significant).

We have previously shown that CPVT-associated RyR2 mutations enhance the propensity for store Ca^2+^ overload-induced spontaneous Ca^2+^ release (SOICR) in HEK293 cells [1, 16, 17, 19]. To assess the effect of the CPVT-associated RyR2 G357S mutation on SOICR, we generated stable, inducible HEK293 cell lines expressing RyR2 WT or the RyR2 G357S mutant. The WT or G357S mutant expressing HEK293 cells were perfused with increasing concentrations of extracellular Ca^2+^ (0–2 mM) to induce SOICR as described previously[16, 17]. The SOICR activity was then monitored by using the fluorescence Ca^2+^ dye Fura-2 AM and single cell Ca^2+^ imaging. The number of HEK293 cells that displayed spontaneous Ca^2+^ oscillations at each extracellular Ca^2+^ concentration was determined and normalized to the total number of cells that responded to caffeine (10 mM) to obtain the fraction of oscillating cells for each condition. The increase in the fraction of oscillating cells in response to elevated extracellular Ca^2+^ concentrations reflects the propensity for SOICR. As shown in Fig 1, WT and G357S-expressing HEK293 cells showed similar fractions of oscillating cells at low extracellular Ca^2+^ concentrations (0–0.3 mM). However, the G357S mutant expressing HEK293 cells showed reduced fractions of oscillating cells at higher extracellular Ca^2+^ concentrations (0.5–2 mM) as compared with WT-expressing HEK293 cells. At 2 mM extracellular Ca^2+^, the fraction of oscillating G357S cells is 47.5 ± 2.5%, significantly lower than that of oscillating WT cells (65.3 ± 4.0%). Hence, unlike most of the CPVT-associated RyR2 mutations, the G357S mutation reduces the maximum fraction of HEK293 cells that show SOICR, but does not appear to enhance the propensity for SOICR in HEK293 cells at low extracellular Ca^2+^ concentrations.

### The G357S mutation decreases both the activation and termination thresholds for SOICR

**Fig 2:**
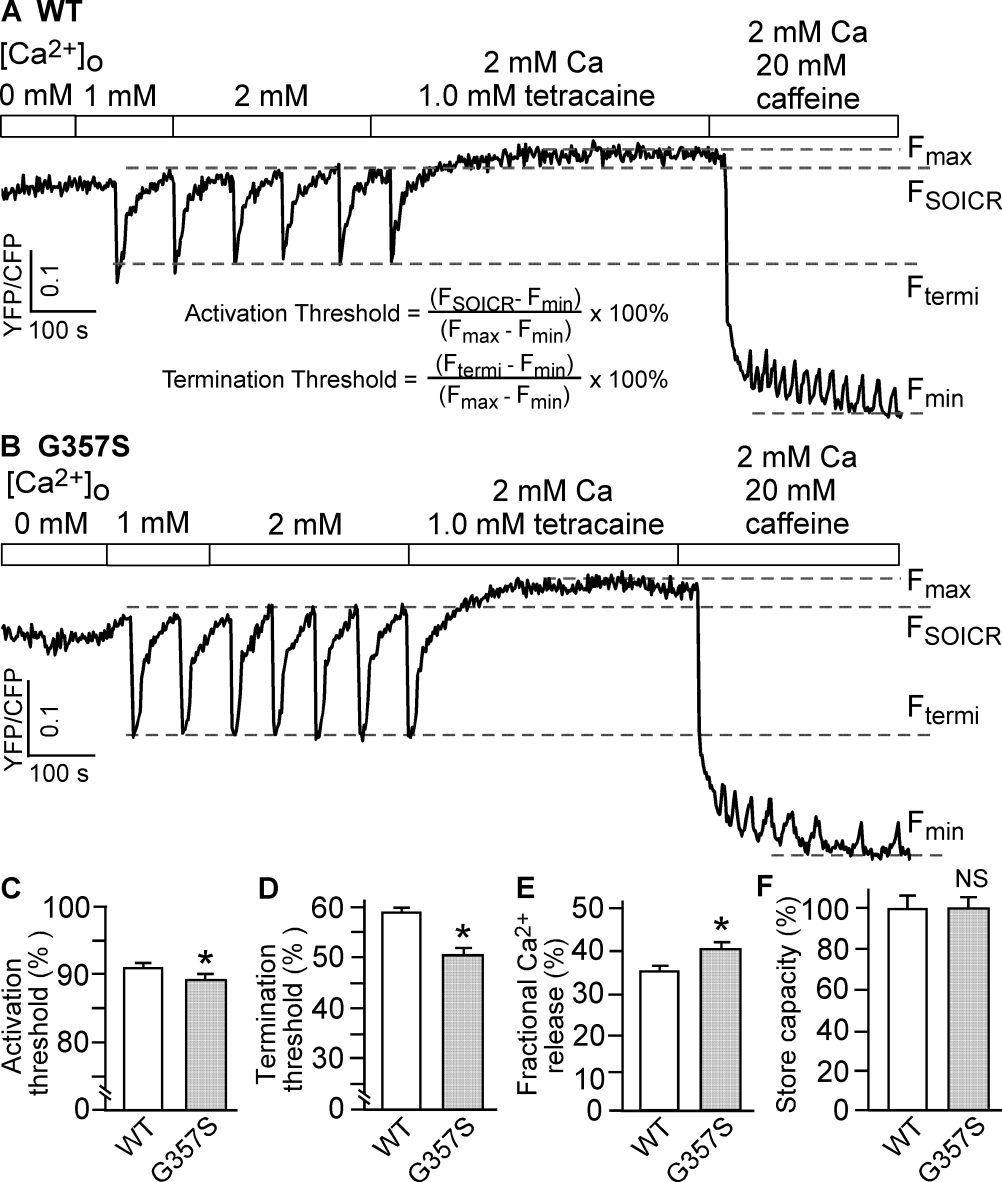
The G357S mutation decreases both activation and termination thresholds for SOICR. Stable, inducible HEK293 cell lines expressing RyR2 WT (A) or the G357S mutant (B) were transfected with the FRET-based ER luminal Ca^2+^-sensing protein D1ER and induced using tetracycline before the experiment. The cells were perfused with KRH buffer containing increasing levels of extracellular Ca^2+^ (0–2 mM) to induce SOICR. FRET recordings from representative cells (total 72 for WT and 77 for G357S) are shown. To minimize the influence by YFP/CFP cross-talk, we used relative FRET measurements for calculating the activation threshold (C) and termination threshold (D) using the equations shown in A. F_SOICR_ indicates the FRET level at which SOICR occurs, whereas F_termi_ represents the FRET level at which SOICR terminates. The fractional Ca^2+^ release (E) was calculated by subtracting the termination threshold from the activation threshold. The maximum FRET signal F_max_ is defined as the FRET level after tetracaine treatment. The minimum FRET signal F_min_ is defined as the FRET level after caffeine treatment. The store capacity (F) was calculated by subtracting F_min_ from F_max_. Data shown are mean ± SEM (n = 5–6) (*, p<0.01 vs WT; NS, not significant).

To further study the effect of the G357S mutation on the properties of SOICR in HEK293 cells that show SOICR, we monitored the endoplasmic reticulum (ER) Ca^2+^ dynamics using a FRET-based ER luminal Ca^2+^-sensing probe D1ER [19, 37]. As shown in Fig 2, increasing extracellular Ca^2+^ concentration from 0 to 2 mM induced spontaneous ER Ca^2+^ oscillations in RyR2 expressing HEK293 cells, shown as downward deflections in D1ER signal. SOICR occurred when ER luminal Ca^2+^ reaches the activation threshold level (F_SOICR_) and terminated when ER luminal Ca^2+^ decreased to the termination threshold level (F_termi_). The activation and termination thresholds were calculated as described in Fig 2A, and the fractional Ca^2+^ release (activation threshold–termination threshold) represents the fraction of Ca^2+^ release during SOICR. The G357S mutation significantly reduced the activation threshold (G357S: 90.1 ± 0.4% vs. WT: 93.2 ± 0.3%) (P<0.01) and the termination threshold (G357S: 50.4±1.3% vs. WT: 58.8±0.9%) (P<0.01)(Fig 2B, 2C and 2D). The G357S mutation also resulted in an increased fractional Ca^2+^ release (G357S: 39.7 ± 1.0% vs. WT: 34.4 ± 0.8%) (P<0.01)(Fig 2E). The store capacity of ER luminal Ca^2+^ (F_max_−F_min_) was not significantly different between WT and G357S (Fig 2F) (P > 0.1). Furthermore, SOICR did not occur in control HEK293 cells expressing no RyR2, and that SOICR was not affected by the IP3R inhibitor, xestospongin C [21], indicating that SOICR is mediated by RyR2. These data indicate that the G357S mutation enhances the susceptibility to spontaneous Ca^2+^ release by reducing the SOICR activation threshold, and augments the amplitude of spontaneous Ca^2+^ release by increasing the fractional Ca^2+^ release as a result of delayed SOICR termination.

### The G357S mutation markedly reduces the level of RyR2 expression in HEK293 cells

**Fig 3:**
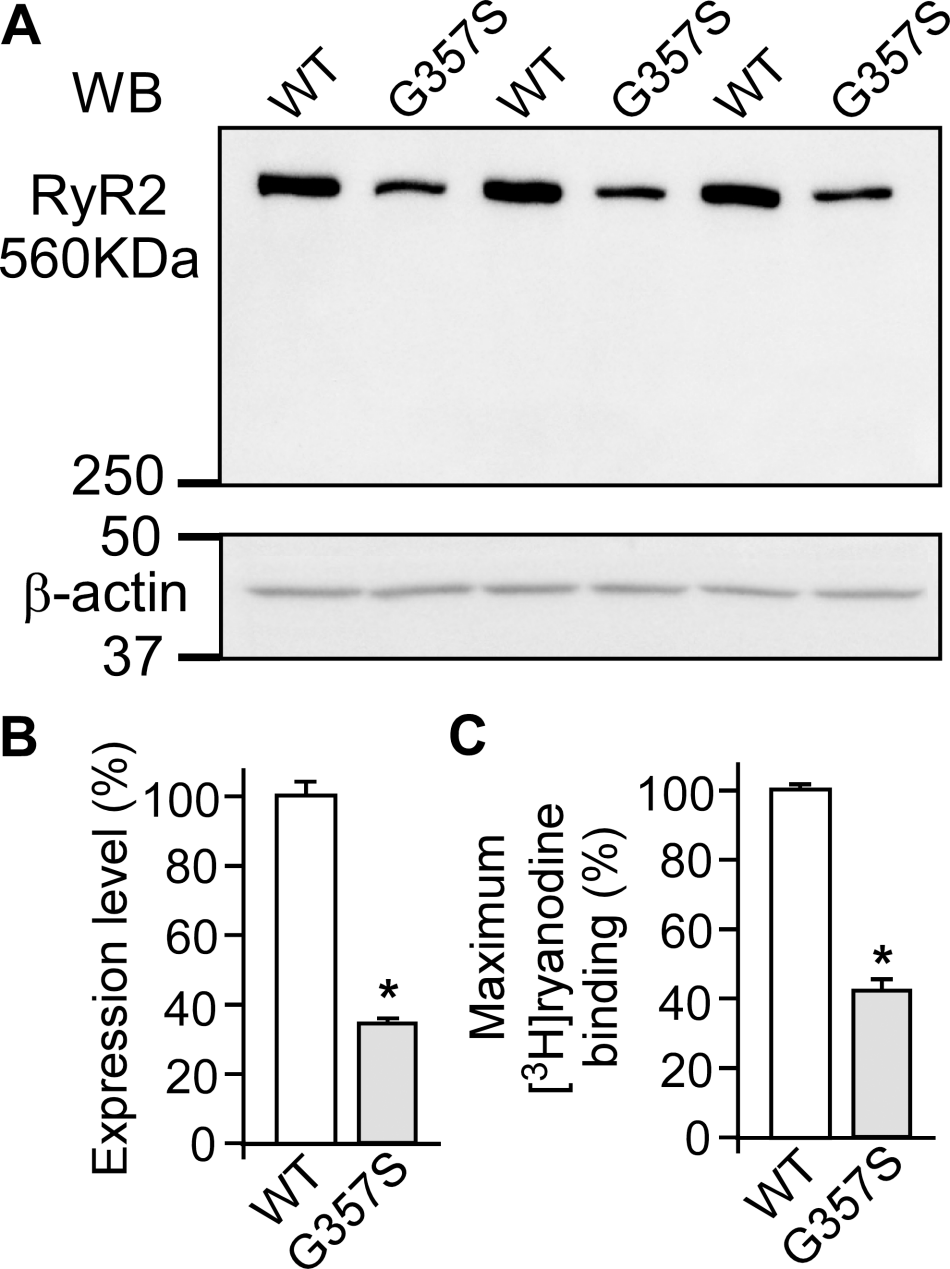
The G357S mutation markedly reduces the level of RyR2 expression in HEK293 cells. Stable, inducible HEK293 cells expressing RyR2 WT or the G357S mutant were collected and lysed after induction for 24 h. The same amount of HEK293 cell lysate protein was used for Western blotting (WB) (A, B) using the anti-RyR antibody (34c) and anti-β-actin antibody or for [^3^H]ryanodine binding in the presence of 500 mM KCl, 5 nM [^3^H]ryanodine, 100 μM Ca^2+^, 2.5 mM ATP, and 2.5 mM caffeine (C). The expression level and the maximum [^3^H]ryanodine binding of the G357S mutant was normalized to that of RyR2 WT. Data shown are mean ± SEM (n = 3) (*, p<0.05 vs WT).

Since the occurrence of SOICR in HEK293 cells depends on the expression of RyR2 [21], the reduced fraction of oscillating cells observed in G357S-expressing HEK293 cells may result from impaired expression of the G357S mutant. To test this possibility, we performed immunoblotting analysis using an anti-RyR antibody to compare the protein levels of WT and the G357S mutant expressed in HEK293 cells. As shown in Fig 3, the expression level of RyR2 in G357S-expressing HEK293 cells is 33.6 ± 1.8% of that in RyR2 WT-expressing cells (Fig 3A and 3B). Since ryanodine only binds to the open conformation of RyRs, [^3^H]ryanodine binding has widely been used for monitoring the activity of the RyR channel. Therefore, we also carried out [^3^H]ryanodine binding to lysates of RyR2 WT and G357S mutant cells to estimate the expression levels of functional WT and the G357S mutant protein. Consistent with the immunoblotting analysis, the maximum [^3^H]ryanodine binding (in the presence of 100 μM Ca^2+^, 2.5 mM ATP and 2.5 mM caffeine) to the G357S mutant cell lysate is 42.5 ± 2.7% of that to the WT cell lysate (Fig 3C). These data demonstrate that the G357S mutation substantially decreases the expression level of the RyR2 protein.

### The G357S mutation does not affect cytosolic Ca^2+^-dependent activation of [^3^H]ryanodine binding

**Fig 4:**
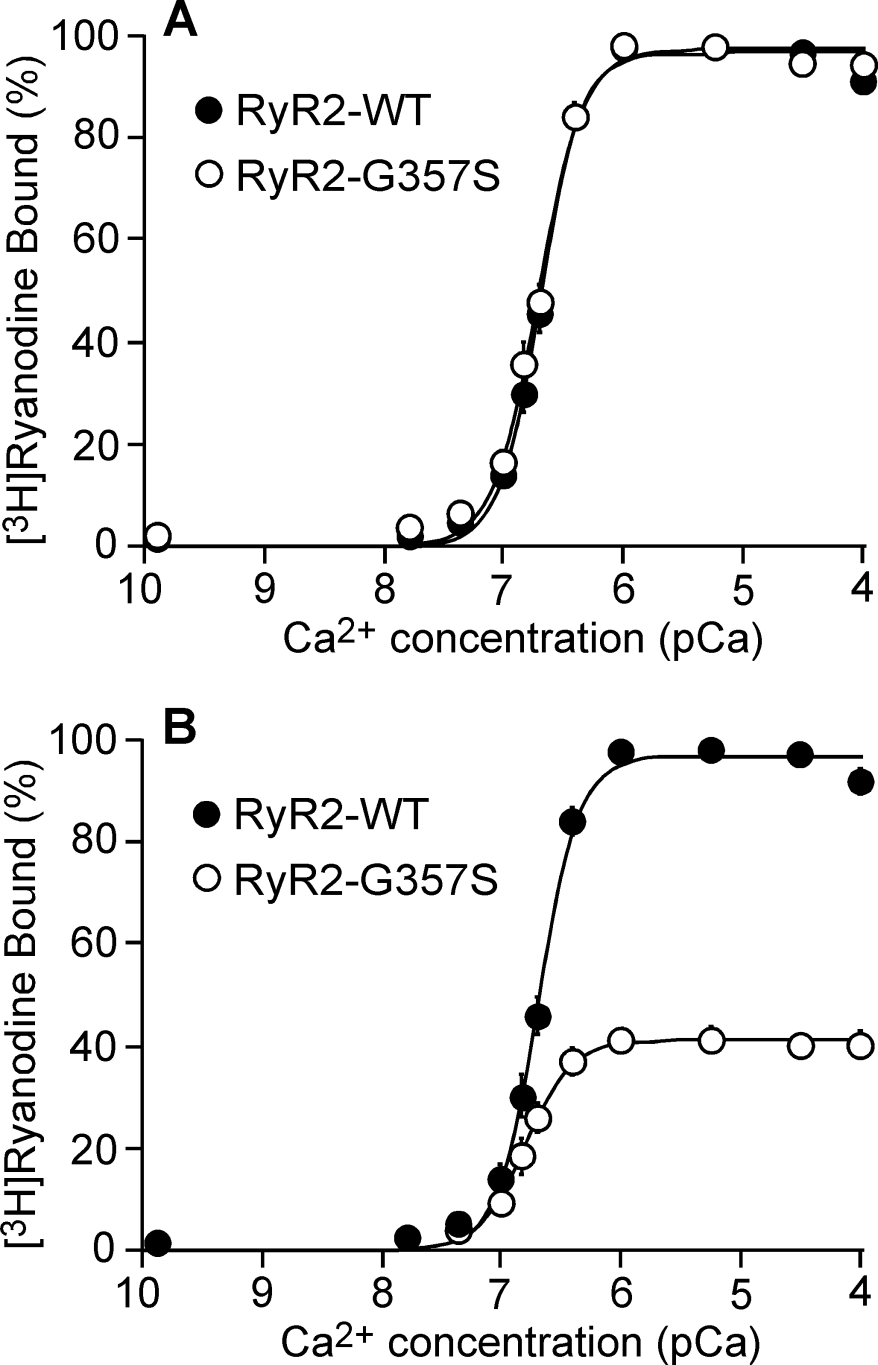
The G357S mutation does not affect cytosolic Ca^2+^ dependent activation of [^3^H]ryanodine binding. [^3^H]ryanodine binding to cell lysates prepared from HEK293 cells expressing RyR2 WT or the G357S mutant was carried out at various Ca^2+^ concentrations (~0.2 nM-0.1 mM), 500 mM KCl and 5 nM [^3^H]ryanodine. (A) [^3^H]ryanodine bound to RyR2 WT or G357S at various Ca^2+^ concentrations was normalized to its own maximal binding (100%). (B) [^3^H]ryanodine bound at various Ca^2+^ concentrations was normalized to the maximal binding to RyR2 WT (100%). The data points shown are mean ± SEM from three separate experiments.

Our previous studies showed that depending on their locations, some CPVT-associated RyR2 mutations enhance the Ca^2+^ dependent activation of [^3^H]ryanodine binding, while other CPVT-associated RyR2 mutations do not [16, 17]. To assess whether the G357S mutation alters the Ca^2+^ dependence of [^3^H]ryanodine binding, we performed [^3^H]ryanodine binding to WT and the G357S mutant in the presence of a wide range of Ca^2+^ concentrations. As shown in Fig 4, the cytosolic Ca^2+^ dependence of [^3^H]ryanodine binding to the WT and G357S mutant was indistinguishable with an EC50 value of 0.21 ± 0.01 μM for WT and 0.20 ± 0.01 μM for G357S (P > 0.1) (Fig 4A). Consistent with those shown in Fig 3, the maximum [^3^H]ryanodine binding to the G357S mutant was significantly reduced as compared with the maximum [^3^H]ryanodine binding to the RyR2-WT (Fig 4B). These data suggest that the G357S mutation does not affect the cytosolic Ca^2+^-dependent activation of RyR2.

### The G357S mutation has little effect on cytosolic Ca^2+^-regulated Ca^2+^ release in HEK293 cells

**Fig 5:**
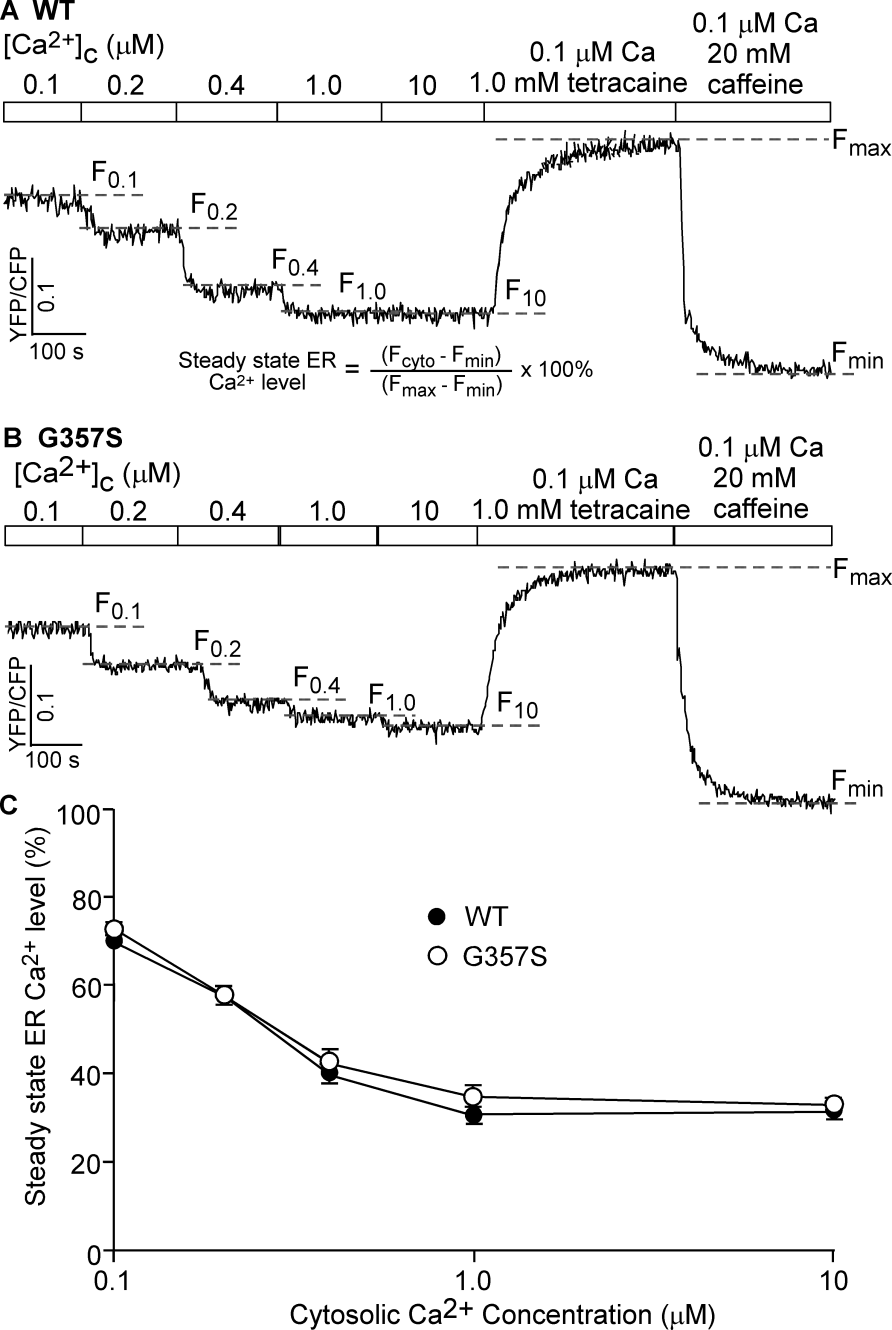
The G357S mutation has little effect on cytosolic Ca^2+^-regulated Ca^2+^ release. Stable, inducible HEK293 cell lines expressing RyR2 WT (A) or G357S (B) were transfected with the FRET-based ER luminal Ca^2+^-sensing protein D1ER and induced using tetracycline. The transfected and induced cells were permeabilized with saponin, washed, and perfused with intracellular-like medium plus increasing levels of free Ca^2+^ (0.1, 0.2, 0.4, 1 and 10 μM). FRET recordings from representative cells (total 89 for WT and 87 for G357S) are shown. To minimize the influence by YFP/CFP cross-talk, we used relative FRET measurements for calculating the steady state ER Ca^2+^ level (defined in A) (C).The dashed lines (F_0.1_-F_10_) indicate the steady state FRET levels after perfusing with each Ca^2+^ concentration (0.1, 0.2, 0.4, 1, or 10 μM). The maximum FRET signal F_max_ is defined as the FRET level after tetracaine treatment. The minimum FRET signal F_min_ is defined as the FRET level after caffeine treatment. The store capacity (D) was calculated by subtracting F_min_ from F_max_. Data shown are mean ± SEM (n = 4–6).

To assess the effect of the G357S mutation on the Ca^2+^ dependent activation of RyR2 in a cellular environment, we determined the level of Ca^2+^ release in permeabilized HEK293 cells triggered by various cytosolic Ca^2+^ concentrations. HEK293 cells expressing the RyR2 WT and G357S mutant were first permeabilized to allow access to cytosolic Ca^2+^. Permeabilized cells were then perfused with various cytosolic Ca^2+^ concentrations (0.1–10 μM). The fractional Ca^2+^ release induced by various cytosolic Ca^2+^ concentrations was monitored by measuring the steady-state ER Ca^2+^ level as described previously [19, 37, 38]. As shown in Fig 5, increasing cytosolic Ca^2+^ concentrations from 0.1 to 10 μM reduced the steady state ER Ca^2+^ level in permeabilized HEK293 cells expressing RyR2 WT (Fig 5A and 5F), which reflects cytosolic Ca^2+^ induced fractional Ca^2+^ release from the ER Ca^2+^ store. The steady state ER Ca^2+^ level at each cytosolic Ca^2+^ concentration in the G357S mutant expressing HEK293 cells is indistinguishable from that in WT-expressing cells. Thus, consistent with the [^3^H]ryanodine binding data, the G357S mutation does not affect the cytosolic Ca^2+^ dependent activation of RyR2.

### The G357S mutation reduces the stability of the N-terminal region of RyR2

**Fig 6:**
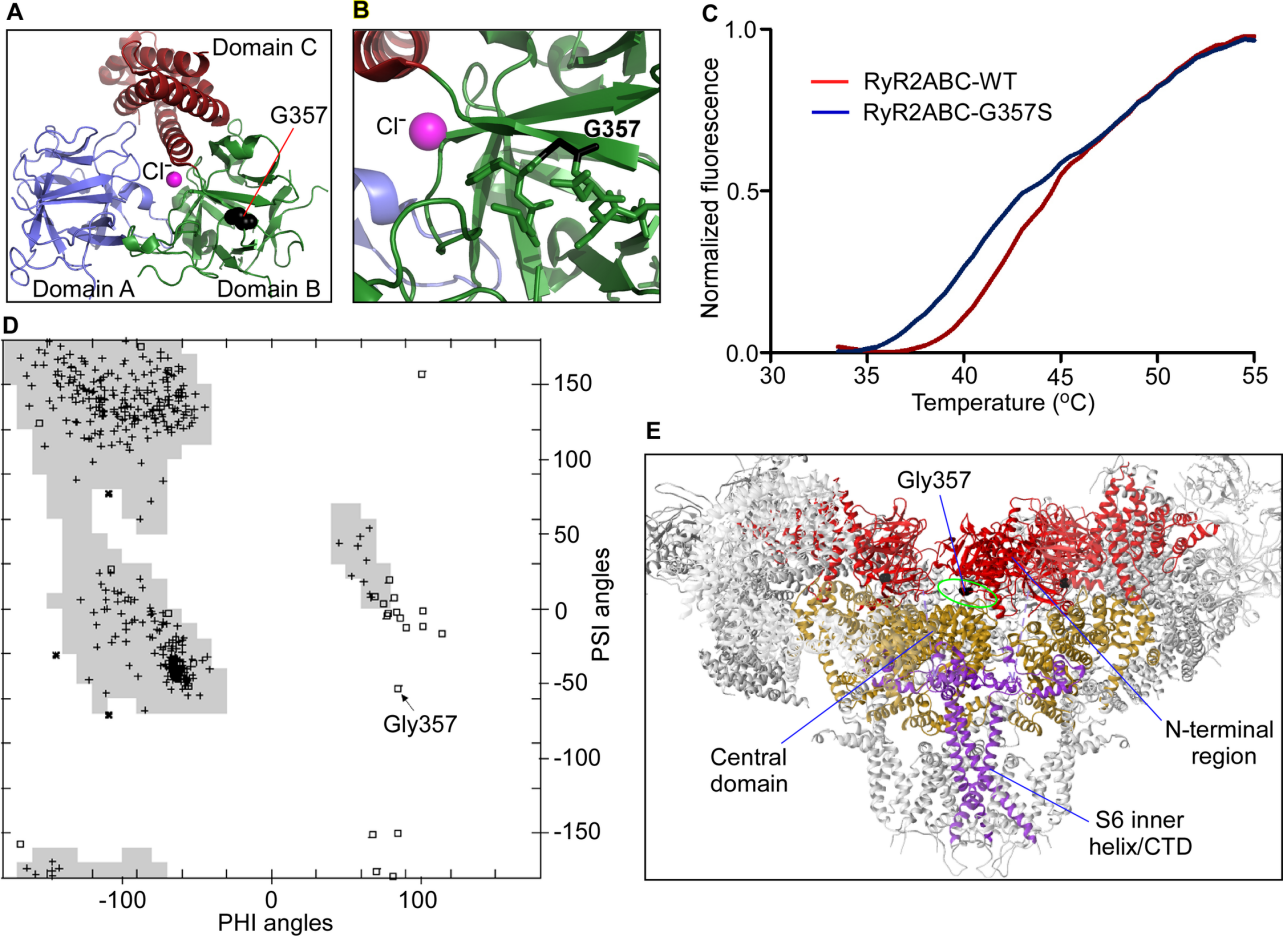
The G357S mutation reduces the stability of the ABC domains of RyR2. **A.** Crystal structure of the mouse RyR2ABC (residues 1–547), showing three domains and a central chloride anion that links the three domains together. **B**. Details showing G357S. The backbone conformation is allowed for glycine, but not serine. **C.** Thermal melting curves indicating thermal stability of WT RyR2ABC vs. G357S RyR2ABC. The melting temperatures, obtained by taking the maxima of the first derivatives, are 42.8°C ± 0.17°C for RyR2ABC WT and 39.8°C ± 0.17°C for RyR2ABC G357S (errors indicating SEM). Statistical significance was determined using an unpaired two-tailed t-test with a 95% confidence level, with P = 0.0002. **D.** Ramachandran plot for the RyR2ABC crystal structure, with backbone phi angles on the X-axis, and psi angles on the Y-axis. The shaded areas indicate preferred regions for non-glycine residues. Glycine residues are indicated with squares, with G357 indicated by an arrow. **E.** Cryo-EM structure of RyR [42, 43], showing the location of the corresponding glycine. One subunit, at the ‘front’ of the viewer, has been left out for clarity. The N-terminal region, the Central domains, and the S6 inner helix bundle with the COOH-terminal domain (CTD) are indicated. The equivalent of G357 in RyR1 is near the interface between the N-terminal region and the Central domain.

Since the G357S mutation reduces RyR2 protein expression (Fig 3), we wondered whether this effect is intrinsic to the N-terminal region where the mutation is located. We expressed the N-terminal region of RyR2 (RyR2ABC) containing the G357S mutation as a fusion protein with His-tag and maltose binding protein (MBP), removed the tags and purified it to homogeneity (Fig 6A and 6B). Fig 6C shows thermal melting curves using a Thermofluor. There are two transitions in the thermal melt for the G357S mutant with the first transition exhibiting a melting temperature significantly lower than that of the RyR2ABC-WT (RyR2ABC-G357S: 39.8 ± 0.17°C vs RyR2ABC-WT: 42.8 ± 0.17°C, P = 0.0002). These results indicate that the G357S mutation has an intrinsically destabilizing effect on the RyR2ABC. In the context of the full-length RyR2, the G357S mutation is close to an interface with the Central domain that encompasses disease hot spot 3 [1, 42, 43] (Fig 6E). Within the individual RyR2ABC domains, G357 is exposed to the surface, and the introduction of a serine side chain would not cause any steric clash with another side chain (Fig 6A and 6B). This may seem at odds with the dramatically reduced expression of the RyR2 protein, but a closer inspection of the backbone dihedral angles explains this. Because of the absence of a C-beta atom, glycine residues can adopt conformations that are not allowed for other amino acids. Fig 6D shows a Ramachandran plot for the RyR2ABC WT protein (PDB code 4L4H). The G357 residue is located in the middle of a disallowed region for non-glycine residues. Substitution by serine would thus cause an intrinsic destabilization of the protein, and we postulate that this underlies the effect on the stability of the G357S mutant protein.

## Discussion

Catecholaminergic polymorphic ventricular tachycardia (CPVT) is thought to be one of the most lethal forms of inherited ion channelopathies [22, 26, 44–47]. However, not all CPVT patients experience this highly lethal phenotypes. Recently, Wangüemert et al. [27] described a large family of more than 1400 members with multiple cases of CPVT and sudden cardiac death. Surprisingly, many G357S mutant carriers lived beyond the age of 50 and some even to their 70–80’s. The mechanism underlying this variable phenotypic expression is unknown.

Understanding the molecular and cellular defects of the RyR2 G357S mutation may provide some clues to the reduced penetrance of CPVT in this large family. Wangüemert et al. [27] showed that the functional impact of the G357S mutation requires sympathetic activation, and suggested that this depencence on beta-adrenergic stimulation may underlie the incomplete penetrance of the disease in this family. In the present study, we determined the impact of the G357S mutation on RyR2 function and expression. We found that the N-terminal G357S mutation reduces the activation and termination thresholds for the arrhythmogenic spontaneous Ca^2+^ release (SOICR), similar to the impact of other N-terminal CPVT RyR2 mutations characterized previously [17, 21]. However, unlike other N-terminal CPVT RyR2 mutations, the G357S mutation also substantially decreases the expression level of the RyR2 protein. These effects of the G357S mutation on SOICR thresholds and protein expression likely contribute to the variable disease phenotypes observed in this family.

It is well established that spontaneous Ca^2+^ release (SOICR) can lead to DADs and triggered activity, which is a major cause of CPVT [1, 8–12]. Hence, a reduced threshold for SOICR activation will enhance the occurrence of SOICR. Furthermore, a reduced threshold for SOICR termination (or a delayed termination) will increase the fractional SR Ca^2+^ release or the amplitude of SOICR. The increased occurrence and amplitude of SOICR would, in turn, increase the amplitude of DADs and thus the propensity for triggered activity and CPVT. Therefore, the enhanced SOICR activity as a result of the G357S mutation may explain the lethal CPVT phenotypes observed in some of the mutant carriers in this large family.

A functional RyR2 channel consists of 4 subunits that form a tetrameric structure. In patients heterozygous for the G357S mutation, the RyR2 channel is composed of both the WT and G357S mutant. The impact of the mutant on RyR2 function would depend on the ratio of the WT and mutant, which depends on the level of expression of the WT and mutant alleles. In the present study, we found that the G357S mutation substantially reduces the level of expression of the mutant RyR2 protein. Thus, it is possible that the variable phenotypic expression observed in this large family may be due to the reduced expression of the G357S mutant protein. Future studies using patient-specific, induced pluripotent stem cell (iPSC) derived cardiomyocytes will be needed to test this possibility.

We have previously shown that CPVT mutations increase the propensity for SOICR as revealed by the increased fraction of cells that displayed SOICR. However, although the G357S mutation clearly affects the properties of SOICR (i.e. reducing the threshold for SOICR activation and termination, and increasing the fractional Ca^2+^ release), we did not observe an increase in the fraction of cells that displayed SOICR. This is likely due to the markedly reduced level of expression of the mutant protein. Since the occurrence of SOICR in HEK293 cells depends on the expression of RyR2 [21], the reduced level of RyR2 expression would decrease the occurrence of SOICR. Consistent with this view, indeed we observed a decreased maximum fraction of G357S expressing cells that exhibited SOICR. Therefore, the enhanced SOICR activity of the G357S mutant may be masked by its reduced expression when the SOICR activities were measured using a cell population-based assay. However, when the SOICR activities were measured in individual cells that displayed SOICR, it is clear that the G357S mutation increases SOICR activity. On the other hand, the G357S mutation has no significant impact on the cytosolic Ca^2+^ activation of [^3^H]ryanodine binding or cytosolic Ca^2+^ induced fractional Ca^2+^ release in HEK293 cells, suggesting that the G357S mutation has little effect on cytosolic Ca^2+^-dependent activation of RyR2, which may also contribute to the variable disease expression in this family.

The mechanism by which the G357S mutation reduces the protein expression of RyR2 is likely intrinsic to the N-terminal region of RyR2. The mutation lies within a loop connecting two beta strands in a β-trefoil domain (domain B). In a high-resolution crystal structure of the RyR2 N-terminal region, the G357 backbone adopts a conformation that is only allowed for glycine residue. The G357S mutation would thus interfere with the folding of the G357 backbone. Indeed, the G357S mutation also results in decreased thermal stability of the N-terminal region of RyR2 (RyR2ABC only). The equivalent glycine appears close to an interface between the N-terminal domain (disease mutation hot spot 1) and the Central domain encompassing disease mutation hot spot 3 of RyR2 [1, 42, 43]. Importantly, the Central domain has recently been proposed to act as the transducer integrating conformational changes in the cytoplasmic assembly into the channel pore domain of RyR2 that controls the gating of the channel [42, 43, 48]. Hence, the interface between the N-terminal and Central domains may be required for normal allosteric coupling between the N-terminal region and the channel pore. As such, the G357S mutation may interfere with the coupling, explaining its effects on SOICR.

HEK293 cells have been widely used to study the impact of disease-associated RyR mutations on the intrinsic properties of the RyR channel. These studies have revealed important insights into the disease mechanisms of a number of RyR mutations. However, HEK293 cells lack many muscle-specific proteins. Thus, whether the intrinsic defects of the CPVT-linked G357S mutation in SOICR and RyR2 protein expression are manifested in cardiac cells has yet to be determined. Future studies using patient-specific, induced pluripotent stem cell (iPSC) derived cardiomyocytes or mouse models harboring the RyR2 G357S mutation will be needed to address this important question.

In summary, in the present study, we demonstrate that CPVT-associated RyR2 G357S mutation increases the SOICR activity by reducing the thresholds for SOICR activation and termination and increasing the fractional Ca^2+^ release. The G357S mutation also markedly reduces the protein expression of RyR2. The impact of the G357S mutation on both the SOICR activity and protein expression of RyR2 may contribute to the reduced penetrance of CPVT in this large family.

## Abbreviations

CPVT: catecholaminergic polymorphic ventricular tachycardia
RyR2: cardiac ryanodine receptor
SR: sarcoplasmic reticulum
ER: endoplasmic reticulum
SOICR: store overload-induced Ca^2+^ release
DAD: delayed afterdepolarization
PBS: phosphate buffered saline.
